# Sensitive Genotyping of Foodborne-Associated Human Noroviruses and Hepatitis A Virus Using an Array-Based Platform

**DOI:** 10.3390/s17092157

**Published:** 2017-09-20

**Authors:** Beatriz Quiñones, Bertram G. Lee, Todd J. Martinsky, Jaszemyn C. Yambao, Paul K. Haje, Mark Schena

**Affiliations:** 1 U.S. Department of Agriculture, Agricultural Research Service, Western Regional Research Center, Produce Safety and Microbiology Unit, Albany, CA 94710, USA; Bertram.Lee@ars.usda.gov (B.G.L.); Jaszemyn.Yambao@ars.usda.gov (J.C.Y.); 2 Arrayit Corporation, Sunnyvale, CA 94085, USA; toddmartinsky@gmail.com (T.J.M.); Paul@arrayit.com (P.K.H.); Mark@arrayit.com (M.S.)

**Keywords:** food safety, foodborne pathogen, genotyping, hepatitis A virus, microarray, norovirus, pathogen detection, viruses

## Abstract

Human noroviruses (NoV) are the leading cause of human gastroenteritis in populations of all ages and are linked to most of the foodborne outbreaks worldwide. Hepatitis A virus (HAV) is another important foodborne enteric virus and is considered the most common agent causing acute liver disease worldwide. In the present study, a focused, low-density DNA microarray was developed and validated for the simultaneous identification of foodborne-associated genotypes of NoV and HAV. By employing a novel algorithm, capture probes were designed to target variable genomic regions commonly used for typing these foodborne viruses. Validation results showed that probe signals, specific for the tested NoV or HAV genotypes, were on average 200-times or 38-times higher than those detected for non-targeted genotypes, respectively. To improve the analytical sensitivity of this method, a 12-mer oligonucleotide spacer sequence was added to the capture probes and resulted in a detection threshold of less than 10 cRNA transcripts. These findings have indicated that this array-based typing sensor has the accuracy and sensitivity for identifying NoV and HAV genotypic profiles predominantly linked to food poisoning. The implementation of this typing sensor would thus provide highly relevant and valuable information for use in surveillance and outbreak attribution.

## 1. Introduction

Human noroviruses (NoV) are a leading cause of sporadic and epidemic gastroenteritis in pediatric and adult populations and are associated with most of the foodborne outbreaks worldwide [1,2]. Most recent estimates of disease burden in the USA have indicated that NoV are responsible for 19–21 million illnesses annually with high estimates of 71,000 hospitalizations and 800 deaths [1,3]. These non-enveloped viruses are composed of single-stranded, positive-sense RNA and belong to a genetically diverse group in the family *Caliciviridae* [4]. Virus infections can be transmitted through different routes, including person-to-person contact, exposure to aerosolized vomitus from an infected individual, and contaminated surfaces, water or food [1,5]. Foodborne transmission vehicles include primarily ready-to-eat foods and mollusks, when served raw or undercooked [6]. Fresh produce, mostly leafy greens and fruits, have also been implicated as relevant food vehicles of NoV infections [6].

The molecular characterization of NoV revealed a genome that is 7.5–7.7 kb in length and composed of three open reading frames (ORF). ORF1 codes for a polyprotein that is cleaved into several non-structural proteins used for viral reproduction. ORF2 and ORF3 encode a major and minor structural protein, respectively, and these proteins are then assembled into the virus capsid [4]. The complete amino acid sequence of the major capsid led to the classification into six genogroups, and in particular, genogroup I (GI) and genogroup II (GII) are associated with the majority of illnesses in humans [3,7,8]. In particular, GII viruses are linked to over 80% of outbreak infections when compared to GI viruses. Phylogenetic clustering analyses of the major capsid protein within these genogroups have led to the subdivision into genotypes [7,9,10]. Recent investigations of NoV outbreaks, provided by laboratory-based surveillance networks, revealed a subset of NoV genotypes predominantly linked to the consumption of food products in the USA and Europe [7,11,12]. Among these foodborne-associated genotypes, genotype GII.4 accounted for the most foodborne outbreaks than any other NoV genotype [7,11,12,13], and other non-GII.4 genotypes, GI.3, GI.6, GI.7, GII.3, GII.6, GII.12, showed significantly high associations with foodborne transmission in the USA and Europe [7,12].

Another important foodborne enteric virus is hepatitis A virus (HAV), which is a small non-enveloped spherical virus in the family *Piconoroviridae* and consists of single-stranded, positive-sense RNA. HAV is a major health problem globally, and the World Health Organization has estimated 1.5 million HAV clinical cases occur globally each year [14,15]. HAV is considered the most common agent causing acute liver disease worldwide [14,16]. Although HAV hospitalizations have decreased nationally from 0.72 to 0.29 per 100,000 persons over a 10-year period from 2002 to 2011, an increased disease burden has been observed as the proportion of patients over 65 years of age almost doubled [16]. HAV can be transmitted by person-to-person contact and through consumption of contaminated water or foods. Foodborne HAV outbreaks have been associated with ready-to-eat foods, shellfish, and produce such as lettuce, green onions, berries and frozen fruits, strawberries and pomegranate [15,17,18,19,20,21,22]. Although the incidence of HAV is generally considered to be low in the United States, there has been a recent emergence of HAV outbreaks associated with the increasing trade of imported food products [18,19,22].

The HAV genome is about 7.5 kb and consists of a single ORF with three distinct regions (P1, P2 and P3) that translate into a single polyprotein [23]. The P1 region encodes capsid proteins while the P2 and P3 regions encode non-structural proteins involved in virus reproduction. The P1 region in turn is subdivided into VP1 thru VP4, and the P2 region is subdivided into 2A, 2B and 2C. Genetic diversity within the VP1-P2 junction regions have been employed traditionally for the assignments of genotypes and sub-genotypes [23]. Currently, there are six HAV genotypes, of which genotypes I and III are the most prevalent among human isolates, and sub-genotype IA is the most common worldwide [23]. However, HAV sub-genotype IB was linked to the multistate outbreak linked to consumption of imported frozen pomegranates [19]. To meet consumer demands, a continued increase of produce imports is expected in the USA [24,25]; therefore, these recent estimates have heightened the development of rapid molecular assays for accurately typing HAV.

The standard molecular method for NoV or HAV detection, employed for epidemiologic and surveillance studies, involves the amplification of viral RNA by reverse transcription-PCR (RT-PCR) due to the greater sensitivity and ease of application than traditional methods such as immunoassays or electron microscopy [8,15,23,26]. More recently, modified versions of the RT-PCR assay, real-time RT-PCR, have consequently become the preferred method in the last decade for the rapid and sensitive detection of foodborne enteric viruses from clinical, environmental and food samples [3,8,26]. For routine outbreak investigations, the real-time RT-PCR protocols rely on the amplification of variable genomic regions in both NoV and HAV for a general virus classification but require subsequent DNA sequencing to identify the specific genotype/sub-genotype of the NoV or HAV extracted from the tested sample [8,23,26,27]. Given that the accurate identification of virus genotypic profiles would provide highly relevant and valuable information for use in surveillance and outbreak attribution [12], there is a critical need for improved and alternative technologies that can rapidly and accurately detect the causative agent.

The development of alternative molecular-based technologies, such as DNA microarrays, offers viable methods to overcome the limitations of RT-PCR assays, which are limited by the number of targets that can be examined [28]. DNA microarrays enable a high degree of parallelism in the identification of specific genotypes by screening multiple markers simultaneously per single assay [29,30,31,32,33]. Previous reports documented the use of microarrays for the detection of the enteric foodborne viruses [34,35,36,37,38,39,40,41,42]; however, the design strategy for the capture probes in these previous studies failed to effectively discriminate among NoV genotypes or only validated a limited number of foodborne-associated genotypes. In the present study, a focused, low-density DNA microarray was developed and validated in conjunction with a rapid and high-throughput fluorescent method. By employing a novel algorithm for capture probe design, an accurate discrimination of multiple foodborne associated genotypes of NoV and HAV was achieved with a high level of specificity and analytical sensitivity. The findings of the present study revealed that this typing array enables a rapid, simple and simultaneous multiplex detection of enteric virus genotypes associated with foodborne disease.

## 2. Materials and Methods

### 2.1. Design Strategy for the Array Capture Probes

**Figure 1:**
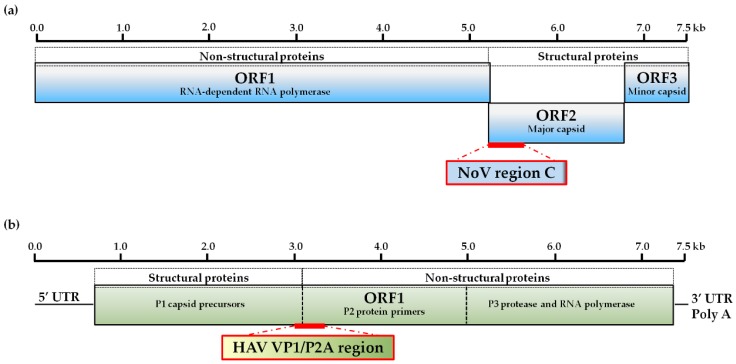
Schematic diagram of the genomic regions in (**a**) NoV and (**b**) HAV targeted by the capture probes in the array-based typing assay.

**Figure 2:**
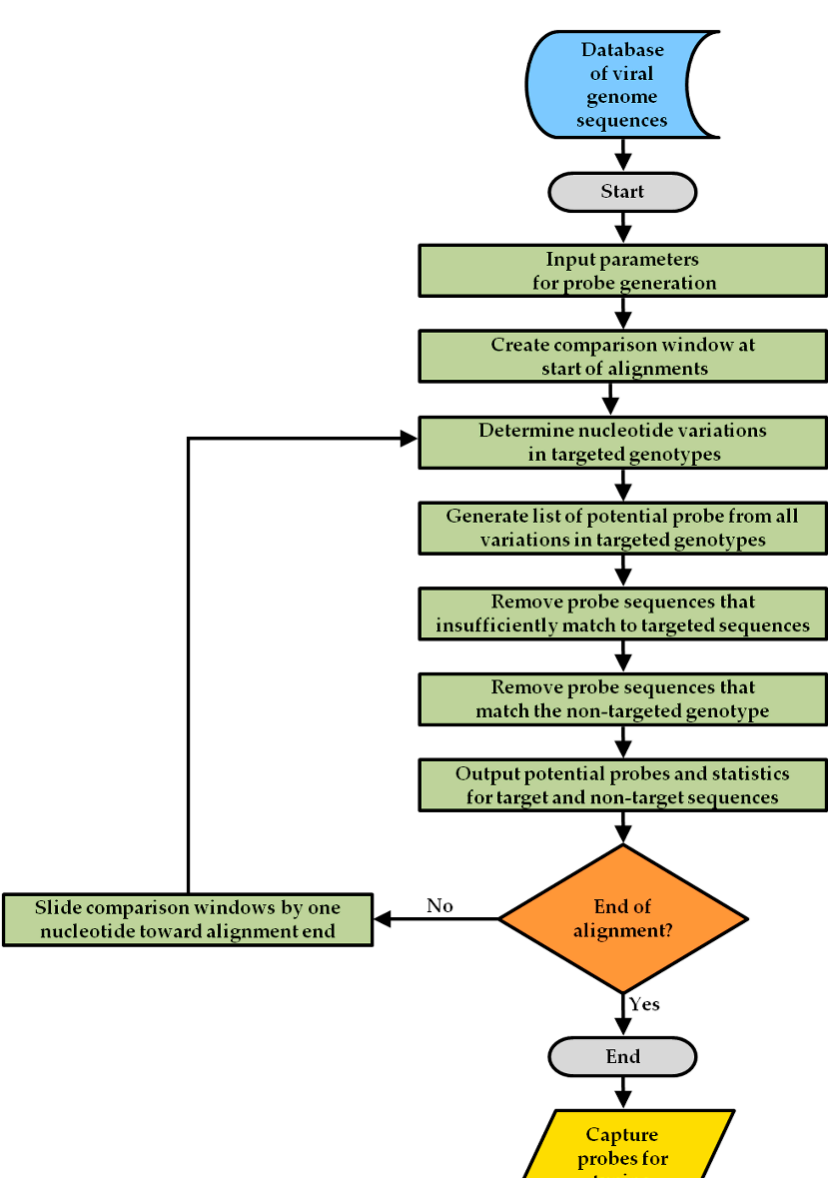
Steps of the VGM algorithm for the design of capture probes, detecting distinct genotypes of NoV or HAV.

For detecting the enteric foodborne viruses, NoV and HAV, capture probes, ranging in length from 25 to 35 bases, were designed to target the variable genomic regions that are commonly used for typing NoV and HAV. As shown in Figure 1, human NoV detection was achieved by designing probes targeting the variable genomic region C, comprised of an overlapping region of ORF1, encoding RNA polymerase, and ORF2 coding for the major capsid protein in both NoV GI and GII [26,43]. Both of these genogroups are responsible for most human disease [3,7,8]. The probe design focused on the typing of twelve NoV genotypes (GI.2, GI.3, GI.4, GI.6, GI.7, GII.1, GII.2 GII.3 GII.4, GII.6, GII.7 and GII.12), most often associated with foodborne illness [7]. For HAV detection, probes targeted the VP1/P2A junction region at the end of the capsid protein and the beginning of the non-structural proteins in ORF1 [23]. The HAV probes detected strains, belonging to the foodborne-associated genotypes IA and IB [23,44]. To achieve the detection of distinct genotypes of NoV or HAV, a novel algorithm called Virus Genome Matching (VGM) (Figure 2), was developed for the capture probe design [45].

As a starting point in the capture probe design strategy (Figure 2), a genome sequence database for NoV was built from sequences deposited in CaliciNet, a NoV outbreak surveillance network [7,46]. The genome database for HAV was obtained by searching online resources with the National Center for Biotechnology Information [47]. The VGM algorithm reads a file containing an alignment of these genome sequences to search and generate specific target probes with a defined high percentage match to all known variations in the sequence of the targeted genotype (Figure 2). The algorithm takes input parameters, including the size of probe in oligonucleotides, the minimum percentage match to target sequences, the maximum percentage match to non-target sequences, and the number of degenerate nucleotides in probes. For the analysis, a window is created to scan the sequences using the input parameters for generating all potential probes.

Given that each virus genotype may have a number of nucleotide variations among different strains, the VGM algorithm was developed not to design a set of probes that would have an exact match to each genotype variant, but rather to design a subset of probes that would hybridize to all possible genotype variants even if there was not an exact base pair match. In addition, the VGM algorithm was designed so that probes would not hybridize to non-target genotypes showing a sequence similarity below 80–85% to the target genotype. Cut-off values of 20% divergence for NoV have been used as a putative genotype boundary to discriminate between genotypes [48]. The algorithm checked the candidate probes against the non-target genotype sequence alignment to exclude any probe that matched to a non-target sequence above the cut-off value defined for non-target sequences. The algorithm stored the candidate probes that sufficiently detected the targeted sequences and that discriminated against the non-targeted sequences. The analysis of match scores shifted the window one nucleotide at a time, and this process was continuously repeated until the end of the alignment was reached. As a final step, to verify for a lack of secondary structures that would prevent the hybridization to the target amplicon, the selected capture probes were then subsequently tested with Geneious 6.1.8 software (Biomatters, Ltd., Auckland, New Zealand) with the ViennaRNA Package [49], implementing the microarray hybridization temperature and energy model [50].

### 2.2. Spacer Sequence Selection

Previous studies have documented maximal lengths of optimal spacer sequence to be about 10–18 oligonucleotides [51,52]. To identify a nucleotide spacer sequence that can improve the hybridization efficiency, candidate spacers were selected in silico from a pool of 1.7 × 10^7^ possible 12-mer oligonucleotides. To minimize the melting temperature (Tm), the three hydrogen-bond G and C nucleotides were removed from consideration, leaving the 2 hydrogen-bond A and T nucleotides in a subsequent list of 8.4 × 10^6^ 12-mer. The AT-content 12-mer were screened using Primer Premier Version 6.22 software (Premier Biosoft, Palo Alto, CA, USA) to eliminate oligonucleotides containing repeated dinucleotide sequences, single base A or T runs of 4 nucleotides or greater, predicted secondary structure (hairpins and self dimers), basic Tm values of 25 °C or greater, and base-stacking Tm values of 13 °C or greater. The selected spacer sequences were then further tested for a lack of hybridization to any documented sequences by using the Basic Local Alignment Search Tool (BLAST) function [47,53,54], and spacer sequences that matched a documented sequences with an *E*-value less than 25 were discarded [55]. A total of 96 candidate 12-mer were selected and evaluated for oligonucleotide synthesis efficiency and hybridization signal strength on microarrays. The 12-mer that met all of the design and performance criteria were chosen as an optimized spacer set. These analyses led to the identification of a 12-bp spacer sequence (5′-TATTAAATAATA-3′), which was added to the 5′ end of the capture probes after the 5′-amino-C6 linker modification for covalent binding to the slide surface.

### 2.3. Virus RNA Sample Preparation and Amplification

NoV RNA was extracted from reference human stool suspensions and were kindly provided by Dr. J. Vinjé with the National Calicivirus Laboratory, Centers for Disease Control and Prevention (CDC, Atlanta, GA, USA). The samples from NoV outbreaks were collected through CaliciNet, a national NoV outbreak surveillance network of laboratories in the United States, coordinated by the CDC [46]. The extracted RNA samples for NoV were facilitated thru the reagent exchange program as a partner institution with the USDA-NIFA Food Virology Collaborative (North Carolina State University, Raleigh, NC, USA) [56]. HAV RNA samples were extracted from clinical stool specimens, subjected to RT-PCR following established procedures [57,58,59], and were kindly provided by Dr. G. Vaughan with the Molecular Epidemiology & Bioinformatics Laboratory, Division of Viral Hepatitis, CDC. All clinical stool specimens were obtained from patients suffering typical gastrointestinal symptoms including nausea, vomiting, low-grade fever and non-bloody diarrhea [5,23]. NoV RNA from clinical stool sample were subjected to the standard real time RT-PCR assays to confirm viral presence based on low cycle threshold (Ct) values of real time RT-PCR assays, indicating a higher amount of virus [60]. The estimated median Ct values were 24 and 22 for the CaliciNet samples from symptomatic specimens containing NoV GI and GII genotypes, respectively [60]. Positive specimens were subjected to RT-PCR followed by DNA sequencing to determine the genotype [7,58,60]. All RNA samples were stored at −80 °C until further use.

**Figure 3:**
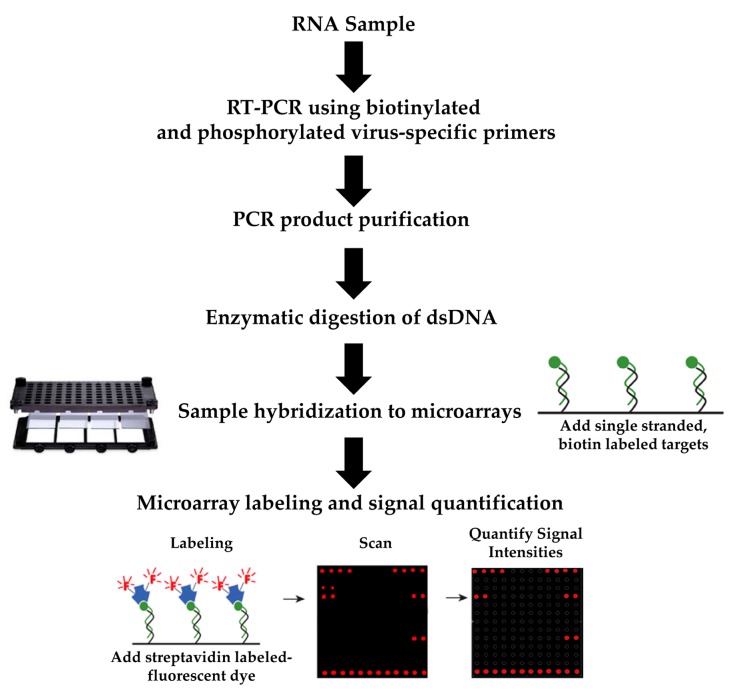
Steps of the array-based method for detecting distinct genotypes of NoV or HAV. The starting material was an RNA sample subjected to RT-PCR, purified, and enzymatically digested to remove the non-complementary strand. The hybridization steps was followed by the microarray labeling and signal amplification and quantification steps. Sample-to-result time is below 8 h.

The detection of NoV and HAV genotypes was achieved by performing a RT-PCR amplification of biotinylated fragments (Figure 3). All sequence-specific primers for the RT-PCR were purchased from Eurofins Genomics (Louisville, KY, USA) with a 5′-phosphorylated linker modification for the forward primers and a 5′-biotin linker modification for the reverse primers, as in previous studies [61,62]. The amplifications were performed using a QIAGEN^®^ One-Step RT-PCR kit (QIAGEN, Valencia, CA, USA) in a 50 μL reaction mixture per the manufacturer’s specifications, except with the replacement of the dNTP mix in the kit with a 20× biotinylated dNTP mix (InDevR, Inc., Boulder, CO, USA). Amplification of a 330-bp fragment, corresponding to NoV genomic region C, in GI was performed with primers G1SKF and G1SKR [43]. Region C amplifications of a 344-bp fragment for GII were performed with primers G2SKF and G2SKR [43]. Amplifications of a 375-bp fragment corresponding to the VP1/P2A junction region in HAV were performed with primers +2934 and −3285, as previously documented [27]. The reaction mixtures were placed in a Dyad Peltier Thermal Cycler (Bio-Rad Laboratories, Hercules, CA, USA), and for NoV amplifications, the following settings were used: 30 min at 50 °C, 15 min at 95 °C, followed by 40 cycles of 45 s at 94 °C, 1 min at 50 °C, 1 min at 72 °C, and a final extension time of 10 min at 72 °C [43]. The cycling conditions for HAV amplifications were 30 min at 50 °C, 15 min at 95 °C, followed by 40 cycles of 20 s at 94 °C, 20 s at 50 °C, 40 s at 72 °C, and a final extension time of 7 min at 72 °C [23,59]. Amplicons were analyzed in 1% agarose gels containing 0.04 μL/mL Gel Red Nucleic Acid Stain (Phenix Research, Candler, NC, USA). RT-PCR amplicons were purified by using the MinElute^®^PCR purification kit (QIAGEN), and the DNA concentration was quantified using a NanoDrop^®^ ND- 1000 spectrophotometer (NanoDrop Technologies, Inc., Wilmington, DE, USA). The amplicon nucleotide sequence was determined by DNA sequencing analysis (Elim Biopharmaceuticals, Inc., Hayward, CA, USA) to confirm the NoV or HAV genotype.

### 2.4. Preparation of In Vitro RNA Transcript Controls

The sources of DNA for preparation of in vitro RNA transcript controls were obtained from RT-PCR amplification of each NoV and HAV genotype using the reagents provided by the QIAGEN^®^ One-Step RT-PCR kit (QIAGEN) to generate products of the NoV region C or HAV VP1/P2A junction, as described in the experimental section above. Each amplicon was cloned into the pCRII-TOPO plasmid vector provided in the TOPO^®^ TA Cloning^®^ Kit (Invitrogen, Carlsbad, CA, USA) and transformed into One Shot™ TOP10 competent *Escherichia coli* cells (Invitrogen), according to the manufacturer’s instructions. Plasmid DNA was extracted and purified with the QIAprep^®^ Spin Miniprep kit (QIAGEN) and sequenced to confirm the orientation and genotype of the insert. For each virus genotype, plasmid DNA was linearized with a restriction enzyme by cutting the plasmid at a site downstream of the insert, and the linearized plasmid DNA was used as a template for the in vitro transcription with either the MEGAscript^®^ T7 Kit or MEGAscript^®^ SP6 kit (Ambion Inc., Austin, TX, USA) [63,64]. Transcripts were treated with TURBO™ DNase (Ambion) and purified by using a MEGAclear™ Transcription Clean-Up kit (Ambion) [63]. The RNA transcripts were dissolved in the Elution Solution (Ambion), and the concentrations were quantified with a NanoDrop^®^ ND- 1000 spectrophotometer (NanoDrop) or by obtaining a fluorometric measurement using Quant-iT™ RiboGreen^®^ RNA Assay Kit (Invitrogen). The RNA transcript solutions were aliquoted and stored at −80 °C until further use.

### 2.5. Microarray Construction and Hybridization

For microarray construction, the oligonucleotide capture probes were purchased with a 5′-amino-C6 linker modification (Eurofins Genomics) to allow covalent binding to the slide surface. Probes were spotted in duplicate at a final concentration of 50 μM on ArrayIt^®^ SuperEpoxy2 microarray slides (Arrayit Corporation, Sunnyvale, CA, USA). As a positive control for the hybridization reaction, a synthetic 24-mer oligonucleotide probe with a 5′-amino-C6 and 3′-biotin modification (InDevR) was spotted at a final concentration of 500 nM [61,62]; this biotinylated control oligonucleotide did not have any sequence homology to any NoV or HAV strain. The microarrays were manufactured with an approximate spot diameter size of 200 μM and a center-to-center spacing of 480 μM (Arrayit). The microarray slides were stored in a desiccator until further use.

To achieve rapid microarray hybridization, amplicons were subjected to a digestion with 15 U of lambda exonuclease (Thermo Fisher Scientific, Waltham, MA, USA) to generate single stranded DNA by digesting the non-complementary strand with a 5′ phosphorylated modification (Figure 3), as described in previous studies [61,62,65]. The exonuclease digestion was performed in a final volume of 25 μL for 30 min at 37 °C, then incubated for 10 min at 95 °C, followed by an immediate addition of 25 μL of 2× Arrayit Hybridization Buffer (Arrayit). The hybridization mixture was applied to the 24-array slides, placed on a 96-well microarray microplate, which was then covered with foil. The mixtures were further incubated for 3 h at 37 °C and 350 rpm using an Array Plate Multi-Well Hybridization Station (Arrayit). All arrays were washed with Hybridization Wash Buffer A, followed by Wash Buffer B, and Wash Buffer C (Arrayit) for 5 min each at 37 °C and 350 rpm. The slides were finally dried by centrifugation using a Microarray High Speed Centrifuge (Arrayit).

### 2.6. Microarray Labeling, Signal Amplification, and Data Quantification

The hybridized microarrays were fluorescently labeled with a 1× solution of 650 nm Detection Reaction Reagent (Arrayit) for 30 min at 37 °C, 350 rpm in the Arrayit^®^ Array Plate Multi-Well Hybridization Station (Arrayit) (Figure 3). Immediately after labeling, the microarrays were washed three times with 1× Detection Wash Buffer (Arrayit) for 1 min at 37 °C and 350 rpm, rinsed with 1× Detection Rinse (Arrayit) for 3–5 s at 37°C and 350 rpm, and dried by centrifugation at 200× *g* for 1 min using a Microarray High Speed Centrifuge (Arrayit). Positive hybridization signals were imaged using an Axon GenePix^®^ 4000B microarray scanner (Molecular Devices, Sunnyvale, CA, USA) at 635 nm wavelength and 10 µM resolution. The acquired images were analyzed using GenePix Pro 6.0 software (Molecular Devices) to calculate the average fluorescence pixel intensity per probe, spotted in duplicate on each replicate array for a total of three replicates per condition tested [66]. Probes were excluded from further quantification analysis if they had an unexpected anomalous spot morphology or were within regions of nonspecific fluorescence [66]. Fluorescent signal intensities were normalized so that the positive biotinylated control spots had an average signal corresponding to 95% of the maximum signal intensity of 65,535 counts. The average signal of all unspecific probes tested in an array was used as the background for each condition examined [35,39]. A detection threshold level was set at 4000 fluorescent counts, corresponding to a threshold value greater than three times the background level of the examined arrays, as described in previous reports [35,39,67].

## 3. Results and Discussion

### 3.1. Validation of Probe Specificity Using the Array-Based Typing Assay for Foodborne Viruses

To evaluate the specificity of the typing method for strains of foodborne-associated enteric viruses, a focused, low-density microarray was constructed using 25–35 mer oligonucleotide probes (Table S1), which were designed with the VGM algorithm (Figure 2). Viral RNA was extracted from reference stool clinical samples containing high-titer viruses, and the purified nucleic acids were amplified with sequence-specific primers and labeled with biotin by RT-PCR. The labeled single-stranded DNA, targeting the genomic region used for typing NoV and HAV (Figure 1), was then hybridized on the microarray. The detection of the signal-amplified biotin labels was quantified by measuring the fluorescent signal values, which were subjected to background correction. The results indicated that a high level of specificity was observed in the detected probe signals when testing a particular genotype for either NoV or HAV (Table 1). In particular, hybridization of labeled nucleic acids from reference GI.2 clinical samples resulted in fluorescent signals with average values of 57,180 counts for probes targeting GI.2; by contrast, fluorescent signals for non-targeted genotypes averaged 191 counts (Table 1).

Similar results were observed when testing nucleic acids extracted from representative clinical specimens for other NoV genotypes. Probe signals specific for the tested genotypes, belonging to GI and GII, were on average 200-times higher than those detected for non-targeted genotypes. Some clinical stool samples, containing virus genotypes GI.4 and GI.7, yielded lower NoV titers when compared to other samples; nonetheless, the ratio of targeted vs. non-targeted probe fluorescent signals was still significantly different and was found to be approximately 40:1 when using this typing array. Although one reference GI.7 clinical sample was available for testing, the validation results, obtained from multiple experiments, indicated a high level of probe signal specificity for detecting this particular NoV genotype (Table 1). A high level of specificity was also obtained when testing RNA extracted from clinical stool samples containing HAV. The specific fluorescent signals detected for probes targeting HAV genotypes IA and IB were 38-times higher than those detected for other probes included on the array (Table 1), and these array-typing results correlated with those obtained by analyzing the samples by DNA sequencing analysis (data not shown). The reference panel was expanded to include the major foodborne pathogens, *Arcobacter butzleri*, *Campylobacter jejuni*, *Campylobacter coli*, *Listeria monocytogenes*, *Salmonella enterica* and *Shiga toxin-producing Escherichia coli* O157 and non-O157. Results showed no specific amplicons after the RT-PCR amplification step. Further quantification analyses indicated average hybridization signals for all GI, GII and HAV specific probes below 250 fluorescence counts, values significantly lower than the detection threshold set at 4000 fluorescent counts.

The findings from these validation experiments clearly indicate that this array-based typing method enables a highly specific detection of multiple NoV and HAV genotypes associated with foodborne illness. Although previous reports documented the use of microarrays for detecting NoV and/or HAV [34,35,36,37,39,40,41,42], these published findings showed either cross reactivity in the identification of some relevant genotypes or only detected and validated a very limited number of genotypes per assay. In summary, the present study is the first report to document the use of a novel algorithm for the capture probe design strategy in conjunction with a focused array platform for accurately identifying twelve NoV and two HAV genotypes in a single assay.

### 3.2. Inclusion of Spacers in the Capture Probe Sequence Significantly Improved Analytical Sensitivity

To accurately determine the sensitivity threshold of this array method, plasmid DNA controls, targeting variable genomic regions in NoV and HAV (Figure 1), were constructed to generate cRNA by performing in vitro transcription reactions from cDNA (see Materials and Methods). A total of 10^2^ copies of cRNA, specific for NoV genotypes GI.2 and GII.12 and HAV genotype IA, were subjected to RT-PCR using sequence specific primers targeting the variable genomic regions in NoV and HAV. For these experiments, a total of 10^2^ cRNA transcripts copies were tested because this amount of cRNA approximated the estimated infectious dose for these enteric foodborne viruses [15,68,69].

**Figure 4:**
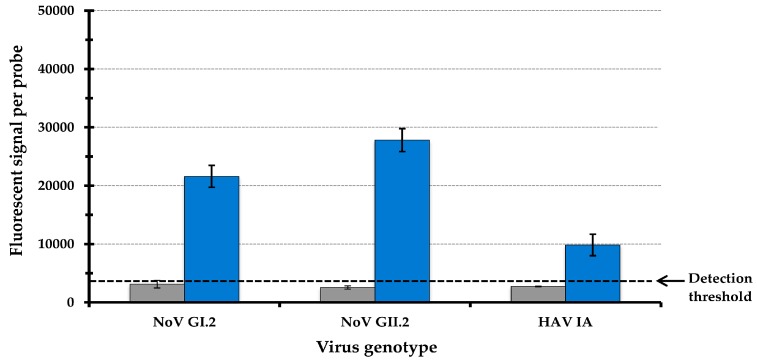
Hybridization signals of the tested NoV and HAV genotypes at 10^2^ cRNA transcript copies in the absence (grey bars) or presence (blue bars) of a 12-mer oligonucleotide spacer sequence attached to the capture probe. The sensitivity detection threshold of the array-based typing method was set at 4000 fluorescent counts and is indicated by the dashed line.

The RT-PCR amplicons were biotin labeled and hybridized to the microarray, and the detected fluorescent signals were quantified. Analysis of the detected microarray signals revealed fluorescent values below the detection threshold (Figure 4, dashed line) when testing 10^2^ cRNA transcript copies for all tested genotypes when testing the capture probes without the addition of a spacer sequence (Figure 4, grey bars). The recorded signal values for NoV GI.2, NoV GII.12 and HAV IA were 3100, 2500 and 2700 fluorescent counts, respectively. When testing higher than 10^3^ cRNA transcript copies, significant average fluorescent values were detected on the hybridized microarray for NoV genotypes GI.2 and GII.12 and HAV genotype IA, corresponding to 12,150, 20,400 and 30,170 counts, respectively.

To improve the analytical sensitivity for detecting NoV and HAV genotypes at 10^2^ transcript copies, which is at or below the estimated infectious dose of these foodborne viruses [15,68,69], a 12-mer oligonucleotide spacer sequence was added to the 5-end of the capture probes. This AT-rich spacer sequence had low binding energy to genomic sequences from enteric viruses. Previous studies have proven a role for spacers in improving the hybridization efficiencies of oligonucleotide probes on DNA microarrays by decreasing steric hindrance between the glass slide and labeled viral amplicons [55,70]. To test for the effect of the spacer sequence on the hybridization efficiencies, 10^2^ copies of NoV GI.2 and GII.12 and HAV IA cRNA transcripts were subjected to RT-PCR, and the amplicons were labeled and hybridized on the array. Our findings indicate that the addition of the spacer sequence to the capture probes resulted in an increase of 6.9-fold and 10.8-fold in the detection of fluorescent-specific signals for NoV genotypes GI.2 and GII.2, respectively (Figure 4, blue bars); the average fluorescent signal values recorded were above 20,000 counts.. A lower but still significant signal increase of 3.6-fold was observed for the detection of HAV genotype IA transcripts with average fluorescent signal values of 9800 counts (Figure 4, blue bars). The effect of the spacer sequence contributed to an increase of the hybridization signals above the detection threshold of this typing array, resulting in the accurate detection of the tested NoV and HAV genotypes at 10^2^ cRNA transcript copies (Figure 4).

**Figure 5:**
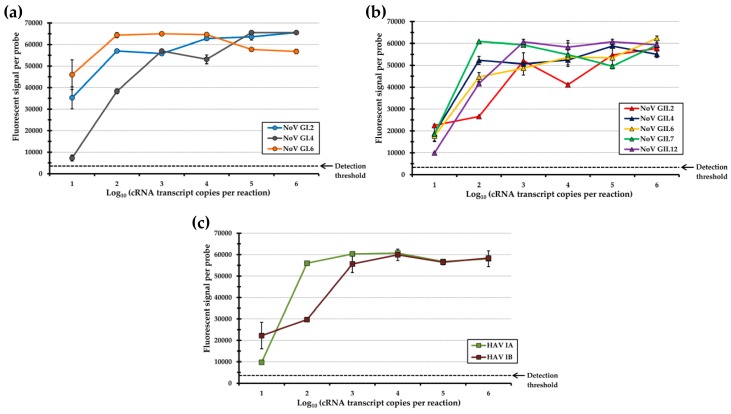
Hybridization signals of various amounts of in vitro cRNA transcripts from (**a**) NoV GI genotypes; (**b**) NoV GII genotypes; and (**c**) HAV genotypes were tested using the array-based typing method. The sensitivity detection threshold of the typing method is indicated by the dashed line.

The improved detection capabilities of this array-typing method, resulting from the use of a spacer sequence attached to the capture probe, prompted additional validation studies for determining the detection limit of other foodborne-associated genotypes of NoV and HAV (Figure 5). Various amounts of in vitro cRNA transcripts were subjected to RT-PCR, and the amplicons were further labeled and hybridized on the array. Quantification of the detected signals on the arrays revealed maximal values for each genotype-specific probe when testing higher amounts of cRNA transcripts, ranging from 10^3^ to 10^6^ copies (Figure 5). When diluting the amounts of tested cRNA transcripts to 10^2^ copies, high fluorescent signals were detected for the capture probes targeting all of the tested NoV and HAV genotypes, and at 10^1^ cRNA transcript copies, the signals were reduced but were still significantly above the detection threshold of this typing method (Figure 5).

The results indicate that the array-based method had a detection limit of less than 10 cRNA transcripts, amounts below the estimated infectious dose for both NoV and HAV [15,68,69]. The improved detection sensitivity for all tested genotypes was at least 25-times better when compared to previous reports also using the array platform for enteric virus identification [36,37,42]. Additionally, the NoV and HAV detection thresholds for all genotypes, using this array-based method, was lower when compared to the reported sensitivities of real time RT-PCR assays [64,71] which are currently the established assay for routine testing by clinical laboratories [8,15]. However, the genogroup-specific oligonucleotide probes employed by real time RT-PCR assays would require subsequent DNA sequencing analysis to further determine most assignments of the specific NoV genotype in the detected strains [7,8,46]. Furthermore, when compared to previous reports using microarray for NoV and HAV detection [34,35,36,37,38,39,40,41,42], the array-based typing method, developed and validated in the present study, proved to be capable of simultaneously detecting a much larger number of relevant genotypes associated with foodborne disease with a high level of analytical specificity and sensitivity. Additionally, the fact that this typing method consists of a low-density, focused array platform would consequently employ reagents and a manufacturing process that are significantly more cost effective when compared to previously documented approaches using high-density microarray platforms [36,37,38,42]. The reduced costs of this focused array platform would enable the adoption of this method by smaller research and surveillance laboratories.

## 4. Conclusions

Enteric foodborne viral pathogens continue to be a significant cause of gastrointestinal illness [2,3]; therefore, there is still a critical need for the development of advanced technologies to accurately identify the causative agent. Specifically, improved methods that can rapidly and accurately detect and characterize virulent strains would be highly relevant and valuable for use in surveillance and attribution. Viral genotypic profiles are required for identifying foodborne outbreaks, implementing preventative measures and recognizing transmission routes [8,12,72,73]. Improvements in the reliability of detection procedures are thus required for routine high-throughput pathogen monitoring. To achieve this goal, the present study employed the use of DNA microarrays as the molecular-based genotyping technology for pathogen identification. The advantage of using the microarray platform when compared to the traditional real-time PCR is the simultaneous detection and genotyping of a much larger number of markers per test sample with a sample-to-result time of less than 8 h [28,62]. Current real time RT-PCR assays require days to provide genotyping information after further amplicon sequencing. Additionally, the array platforms are still less expensive than new sequencing technologies, and arrays are not subject to the challenging analyses of massive amounts of data obtained with the genome sequencing technologies [74,75,76].

The present study is the first one to demonstrate the simultaneous identification of multiple genotypes of foodborne-associated NoV and HAV with a focused, low-density microarray platform. By developing a novel algorithm to improve capture probe design and evaluating methods to maximize the detection signal specificity and analytical sensitivity, the findings from this study have indicated an accurate identification from clinical samples of twelve genotypes of NoV (GI.2, GI.3, GI.4, GI.6, GI.7, GII.1, GII.2 GII.3 GII.4, GII.6, GII.7 and GII.12) and two genotypes of HAV (IA and IB); validation experiments revealed this typing array sensor has a detection sensitivity of at least 10 transcript copies. Given the advantages of using a microarray technology for identifying foodborne viral pathogens, these findings have set a foundation for future studies, aimed at the further adapting emerging detection methodologies in conjunction with the array platform. Further optimization of experimental approaches would still be required to achieve efficient concentration of pathogens, an important issue to address when detecting pathogens present in low concentrations in complex samples [3]. Fully-integrated platforms have been designed with simpler and rapid assays by automating data collection and analysis [8,28]; however, one limitation of these current platforms is that viral pathogens are only identified at the genogroup level, resulting in insufficient strain discrimination. By incorporating cost-effective and portable instrumentation, further research is thus needed to develop and validate these emerging automated platforms that would provide accurate genotyping results and enable strain discrimination and differentiation from a wide variety of samples, recovered from clinical, food, and environmental sources.

## Figures and Tables

**Table 1:** Validation of capture probe specificity using the typing array for detecting multiple foodborne-associated genotypes of NoV and HAV.

Virus Tested	Genotype	Sample ID	Targeted Genotype by the Capture Probes ^1^													
			NoV												HAV	
			GI.2	GI.3	GI.4	GI.6	GI.7	GII.1	GII.2	GII.3	GII.4	GII.6	GII.7	GII.12	IA	IB
NoV	GI.2	2012-1	**59,535**	475	97	75	83	74	156	346	115	85	94	120	84	106
		2012-2	**56,624**	487	209	131	108	104	176	317	179	346	129	135	112	171
		2014-1	**55,381**	797	178	164	155	267	188	330	155	164	132	92	91	239
	GI.3	2012-3	179	**65,535**	246	279	288	147	211	300	253	163	173	172	171	218
		2012-4	198	**36,025**	234	361	229	158	225	319	344	171	198	146	148	253
		2014-23	248	**49,256**	289	268	238	259	272	438	250	320	321	219	259	274
		2014-55	234	**41,789**	371	1291	255	265	304	441	295	424	388	253	289	310
	GI.4	2012-5	2612	469	**65,535**	3146	171	141	730	399	190	215	198	136	140	200
		2012-6	240	371	**9149**	449	148	136	311	427	169	230	190	135	134	193
		2014-35	85	340	**58,587**	670	135	79	212	274	129	105	130	119	103	196
		2014-58	89	377	**46,755**	486	119	78	179	282	106	145	113	105	95	205
	GI.6	2012-7	166	650	674	**62,158**	163	142	252	409	154	351	217	132	133	197
		2012-8	673	468	260	**65,535**	152	164	200	295	146	511	209	132	133	165
		2014-20	134	379	190	**65,535**	360	161	213	324	162	208	183	130	146	193
		2014-41	137	432	198	**65,535**	355	199	220	346	176	191	188	167	157	216
	GI.7	2014-44	199	329	180	176	**19,726**	164	225	288	188	437	260	204	195	224
	GII.1	2012-9	80	274	124	114	106	**16,444**	189	239	108	128	99	153	203	163
		2012-10	153	364	192	907	136	**65,535**	247	334	178	183	228	173	130	156
		2014-18	148	380	172	160	151	**24,253**	248	299	168	163	709	133	242	245
	GII.2	2012-11	71	315	79	77	150	172	**65,535**	235	119	99	156	1562	82	91
		2012-12	78	232	75	78	156	87	**65,535**	377	105	308	383	521	87	93
		2014-48	119	371	154	145	138	125	**30,397**	151	173	132	228	157	138	180
	GII.3	2014-31	85	281	80	90	104	86	216	**65,535**	196	113	114	156	90	99
		2014-39	78	142	81	90	93	85	211	**65,535**	189	111	115	129	93	102
	GII.4 New Orleans	2012-13	150	331	191	250	137	162	303	203	**65,535**	256	241	161	126	163
		2012-14	298	242	336	237	227	211	277	221	**55,210**	245	529	295	284	246
		2014-2	169	370	181	176	154	139	326	204	**33,307**	159	189	560	157	185
		2014-3	133	399	198	159	152	141	336	204	**46,211**	182	412	157	219	239
	GII.4 Sydney	2014-4	153	401	178	163	146	139	336	230	**47,929**	223	619	294	223	226
		2014-14	298	517	206	195	220	181	371	338	**65,535**	305	304	267	305	367
	GII.6	2014-12	73	180	75	79	94	72	187	135	218	**9375**	85	103	81	79
		2014-13	63	236	79	80	133	73	208	332	442	**65,535**	115	103	81	93
	GII.7	2014-30	197	388	63	110	112	69	468	264	104	87	**58,415**	170	90	205
		2014-54	287	376	130	128	122	79	2158	296	598	116	**60,589**	677	101	252
	GII.12	2012-16	167	367	182	647	150	232	350	309	167	664	208	**65,535**	124	147
		2014-6	145	399	176	145	134	123	324	196	197	364	1496	**15,745**	128	174
		2014-43	138	358	170	157	134	124	290	186	145	139	673	**11,201**	134	160
HAV	IA	2014-3	211	444	242	216	206	217	311	333	232	207	297	213	**14,067**	270
		2014-5	166	326	227	207	185	181	325	252	210	185	181	189	**8073**	222
		2014-6	190	417	240	236	191	190	325	305	204	267	228	169	**6370**	235
	IB	1347	204	432	235	221	174	178	315	268	208	190	191	170	312	**4479**
		1357	229	453	264	218	207	180	325	309	224	187	194	174	250	**41,234**
		1373	203	476	245	230	183	176	370	323	219	182	194	175	214	**8418**

^1^ Fluorescent signals detected for genotype-specific probes are shown in bold.

## Supplementary Materials

The following are available online at http://www.mdpi.com/1424-8220/17/9/2157/s1, Table S1: List of oligonucleotide capture probes used in this study.
